# Supplementary material for: Hi-C-constrained physical models of human chromosomes recover functionally-related properties of genome organization
Source: Sci Rep. 2016 Oct 27;6:35985. doi: 10.1038/srep35985 (PMC5081523; doi:10.1038/srep35985)
Supplement: Supplementary Information [file srep35985-s1.pdf]

# Supplementary Figures

## "Hi-C-constrained physical models of human chromosomes recover functionally-related properties of genome organization"

by Marco Di Stefano, Jonas Paulsen, Tonje G. Lien, Eivind Hovig, and Cristian Micheletti

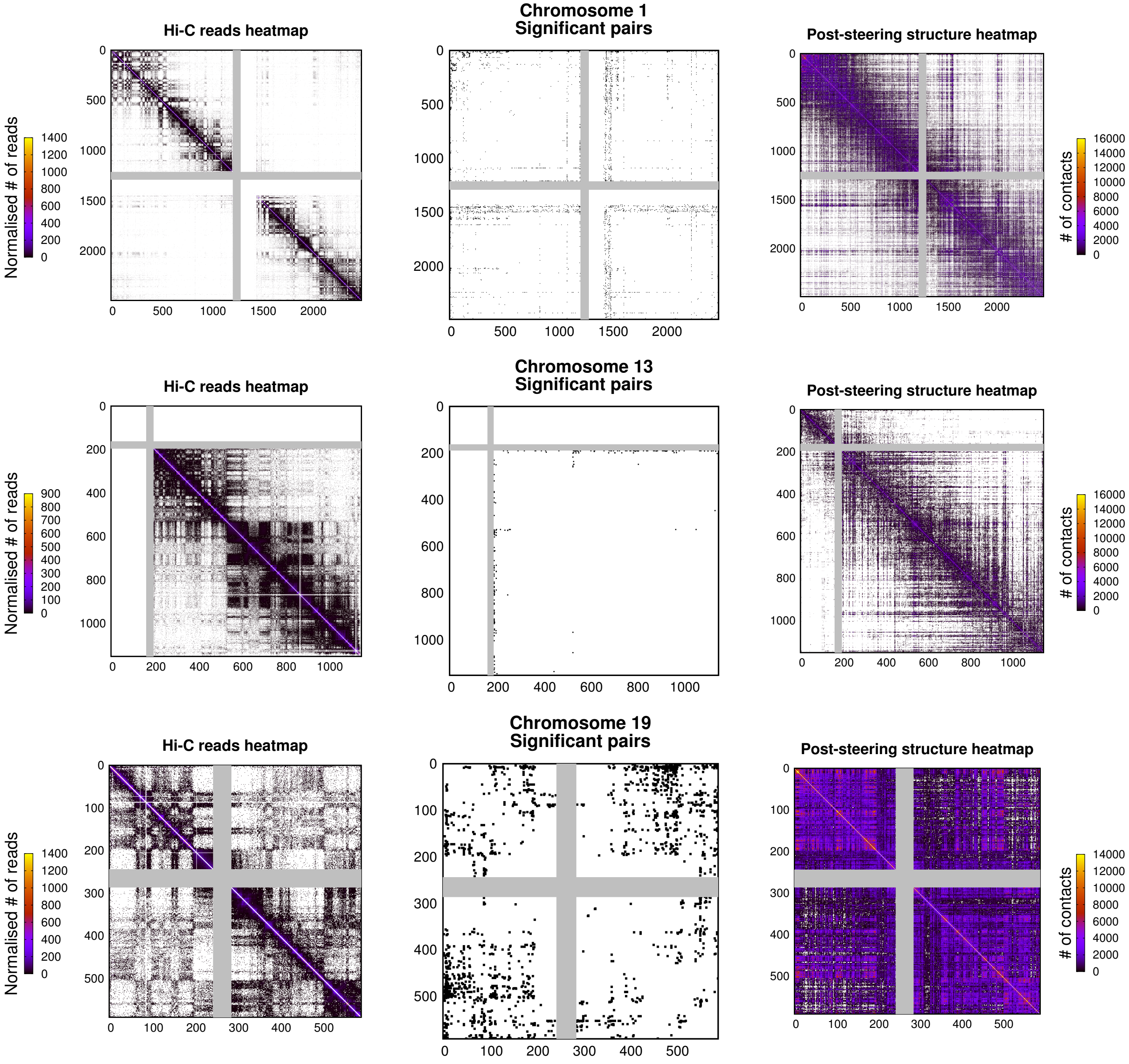

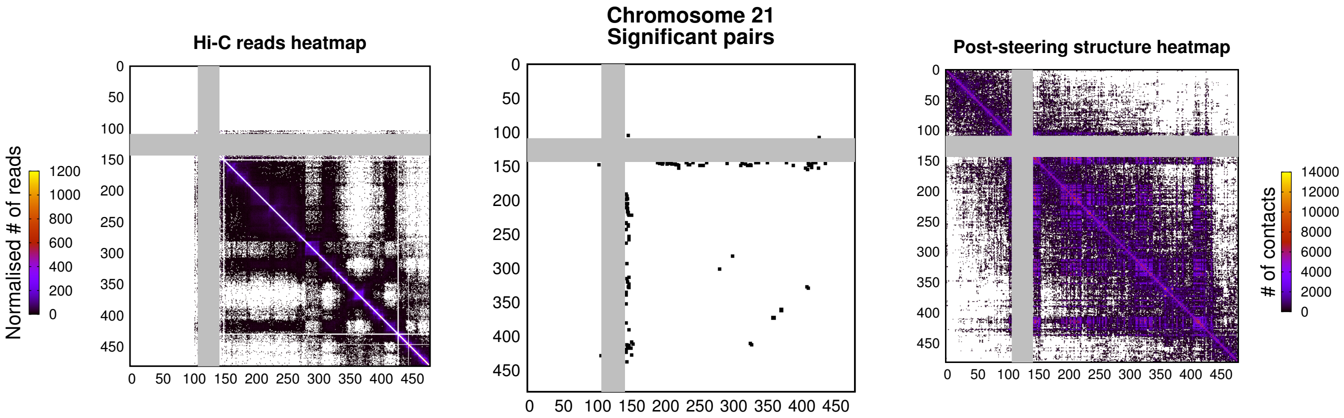


**Supplementary Figure S1**: **Cis-chromosome heatmaps for lung fibroblasts (IMR90) cell line at 100 kilobases resolution.** The panels on the left side show the Hi-C contact propensity maps of human embryonic stem cells from Dixon *et al.* (2012) that have been adjusted for technical biases using the method described in Imakaev *et al.* (2012). Central panels show the subset of statistically significant Hi-C contacts. The dots used for the entries have been magnified for visual clarity. Panels on the right side show the contact map (cutoff 240nm) obtained from the 10 optimally-steered models of IMR90 nuclei with phenomenological initial positioning from Bolzer *et al.* (2005). Only selected chromosomes are represented, namely: chr1 which is the longest, chr21 which is the shortest, chr19 which has the largest sequence-wise density of target constraints, and chr13 which has the smallest one. In all panels, the gray bands mark the centromeric region

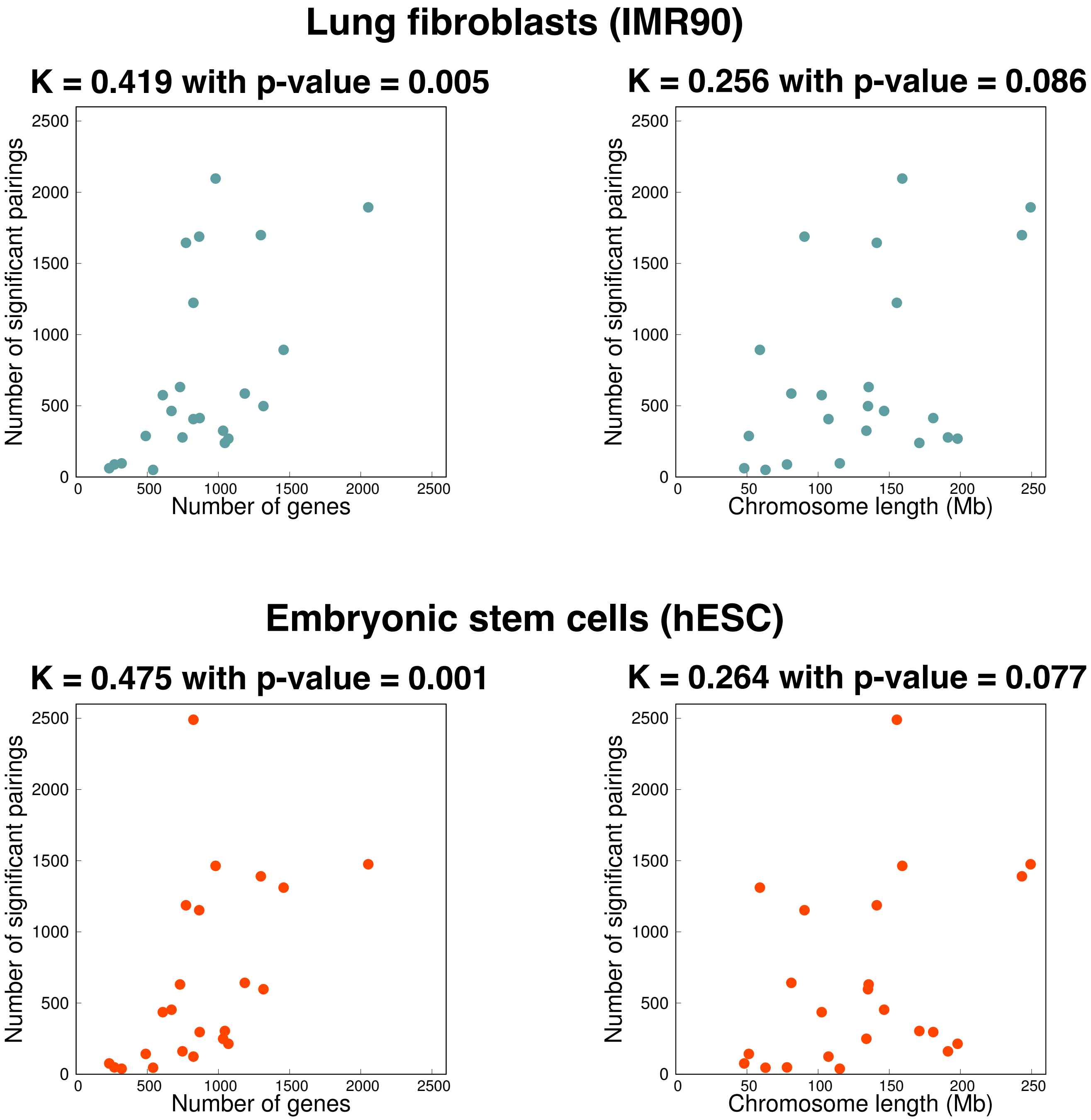


**Supplementary Figure S2**: **Number of significant pairings per chromosome based on the analysis of the data in Dixon *et al* (2012) for lung fibroblasts (IMR90) and embryonic stem cells (hESC).** For both cell lines, the number of significant pairings correlates significantly with the number of genes in the chromosomes ($p - value < 0.005$ of non-parametric Kendall rank-correlation), but only weakly with chromosome length ($p - value > 0.08$).

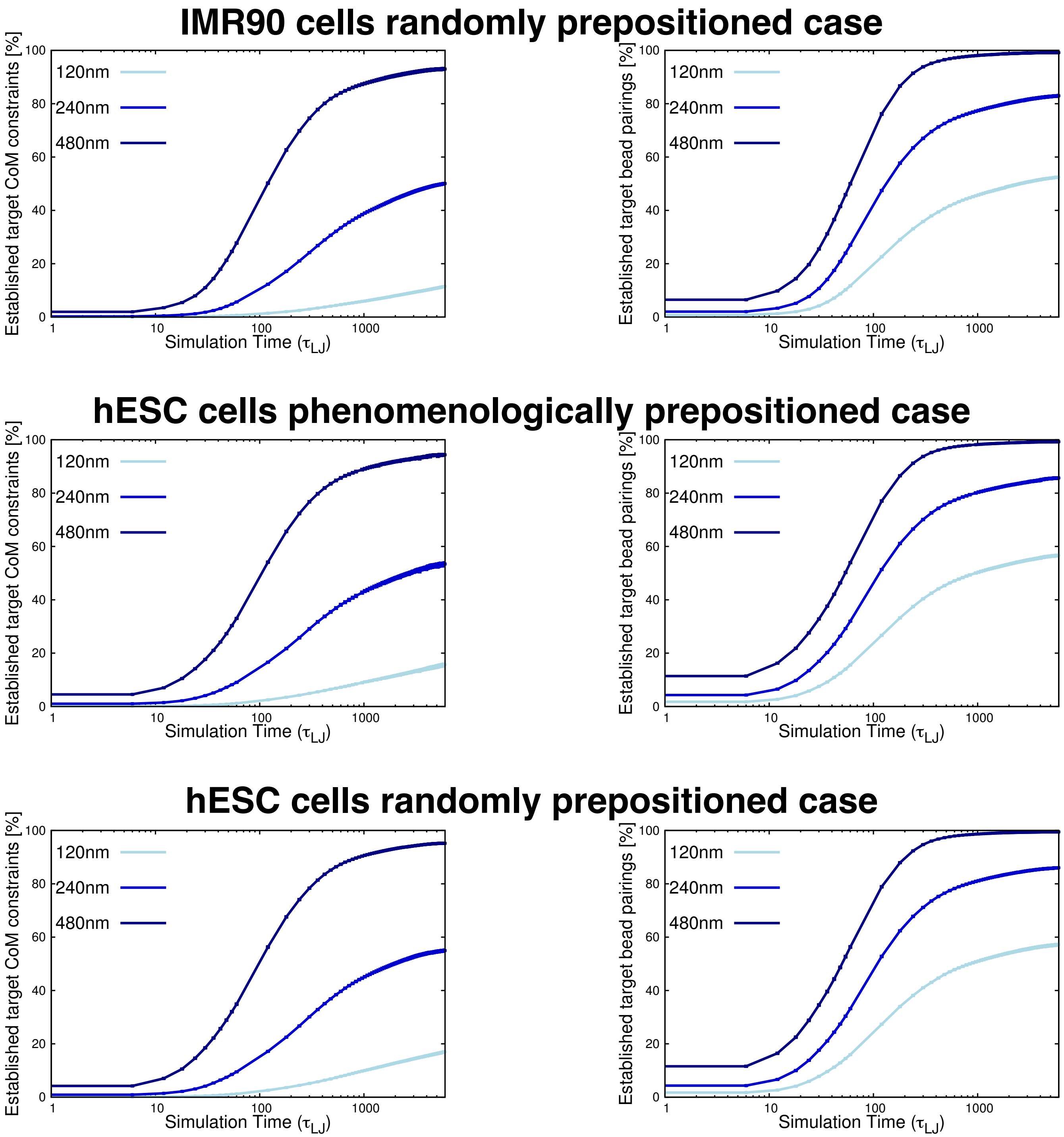


**Supplementary Figure S3**: **Evolution of the satisfied target constraints from the analysis of the data in Dixon *et al* (2012) for different cell lines and chromosome positioning schemes**. The curves show the increase of the percentage of target contacts that are established in the course of the steering dynamics for lung fibroblasts cells (IMR90) and human embryonic stem cells (hESC) nuclei and for different chromosome positioning schemes (phenomenological and random). The plots complement the information provided in Fig. 1 of the main text.

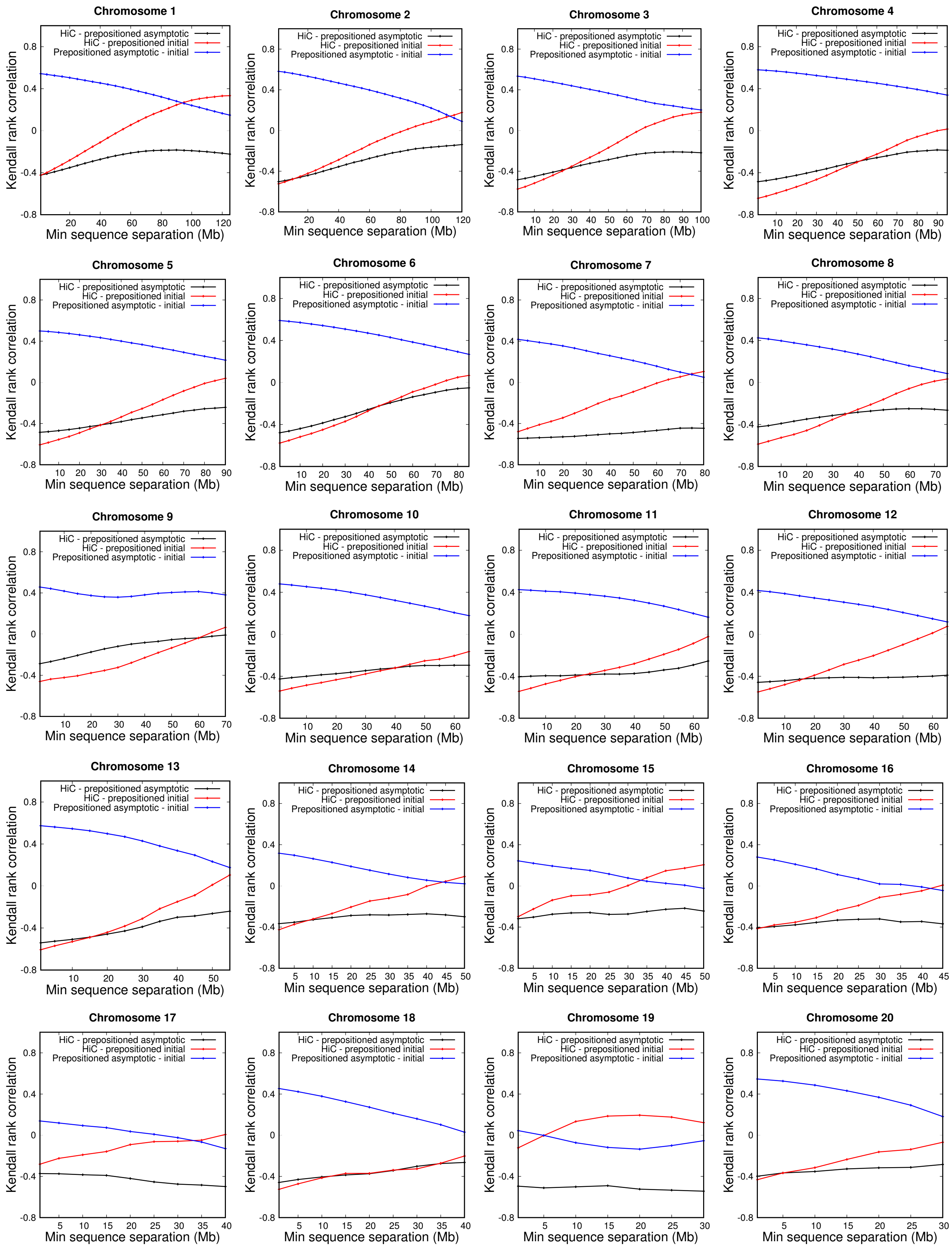
Chromosome 1
Chromosome 2
Chromosome 3
Chromosome 4
Chromosome 5
Chromosome 6
Chromosome 7
Chromosome 8
Chromosome 9
Chromosome 10
Chromosome 11
Chromosome 12
Chromosome 13
Chromosome 14
Chromosome 15
Chromosome 16
Chromosome 17
Chromosome 18
Chromosome 19
Chromosome 20
HiC - prepositioned asymptotic
HiC - prepositioned initial
Prepositioned asymptotic - initial
Kendall rank correlation
Min sequence separation (Mb)

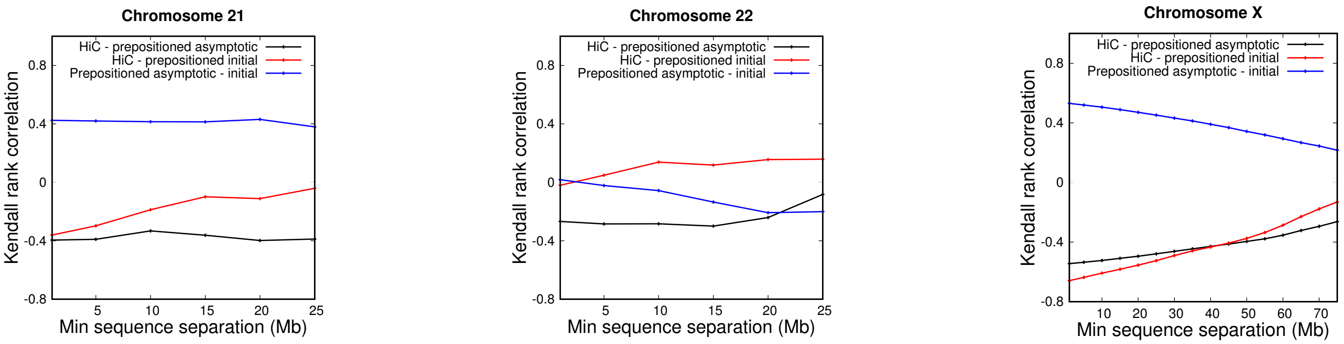


**Supplementary Figure S4**: **Correlation analysis between the Hi-C contact matrices and the models' distance matrices.**. The curves show the *cis*-chromosome Kendall rank correlation coefficients between different matrix pairs considering entries at different minimum sequence separations from 1Mb up to half of the chromosome length. The entries of each *cis*-chromsome Hi-C contact matrix from Dixon *et al.* (2012) are compared with the entries of each model distance matrix in the prepositioned case after steering (black curves) and in the prepositioned case before steering (red curves). For completeness, the two model distance matrices are also compared between them (blue curves). Due to the high number of entries, Kendall correlation coefficients larger than 0.147 in modulus are statistically significant because they have a (two-sided) $p - value$ smaller than 0.05 for the smallest chromosomes, and hence a much lower p-value for the other, longer ones.

### Lung fibroblasts (IMR90) nuclei with phenomenological prepositioning of the chromosomes

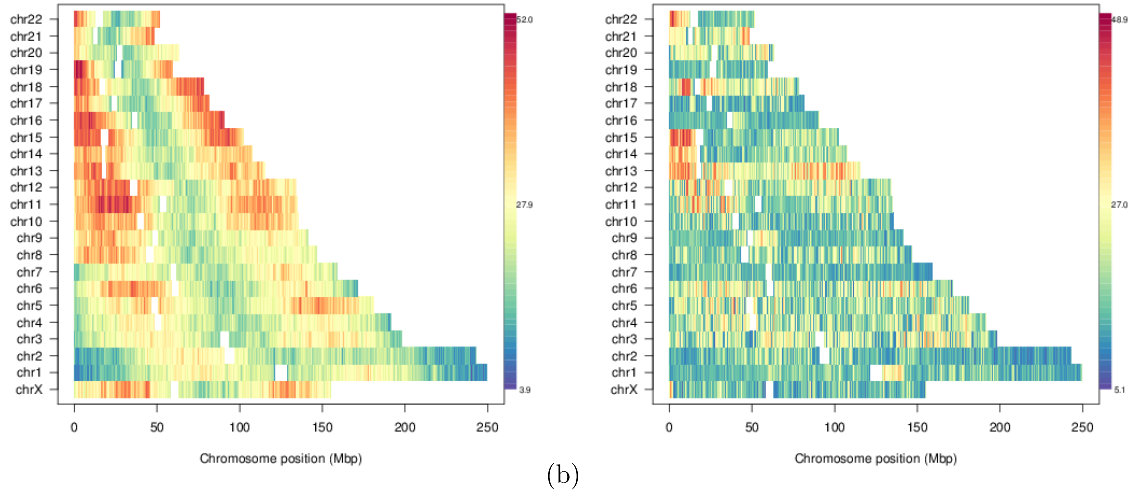


(a) (b)

**Supplementary Figure S5**: **Genome-wide variability of radial bead position.** The panels are based on configurations obtained starting from the phenomenological prepositioning of the chromosomes in Bolzer *et al.* (2005), immediately prior to (a) and after (b) applying the steering protocol appropriate for IMR90 cell line based on the analysis of the data in Dixon *et al* (2012). Numbers indicate the standard deviation of the radial position across the 10 replicate simulations. The plots complement the information provided in Fig. 3 of the main text.

### Lung fibroblasts (IMR90) nuclei with random prepositioning of the chromosomes

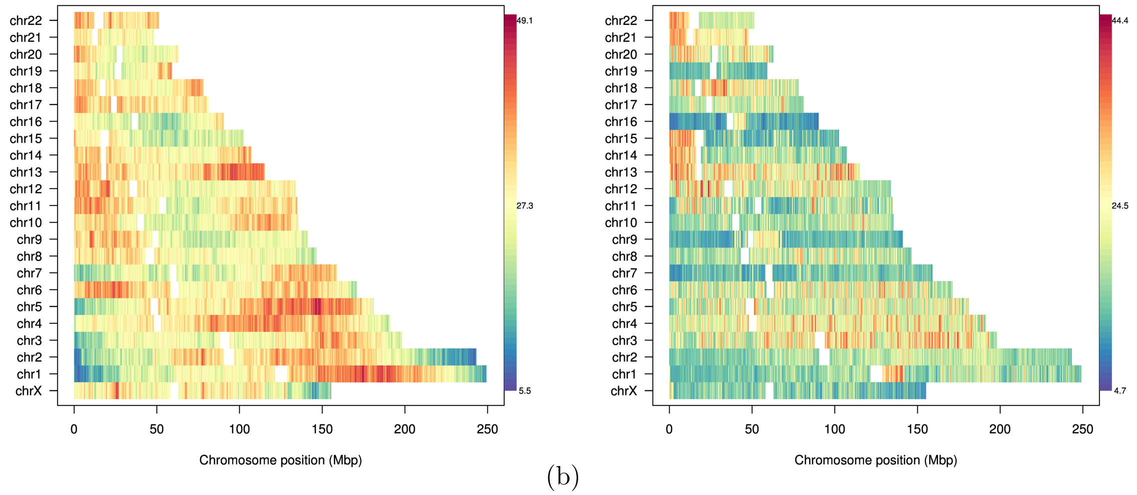


(a) (b)

**Supplementary Figure S6**: **Genome-wide variability of radial bead position.** The panels are based on configurations obtained starting from a random prepositioning of the chromosomes, immediately prior to (a) and after (b) applying the steering protocol appropriate for IMR90 cell line based on the analysis of the data in Dixon *et al* (2012). Numbers indicate the standard deviation of the radial position across the 10 replicate simulations.

**Embryonic stem cells (hESC) nuclei with phenomenological prepositioning of the chromosomes**

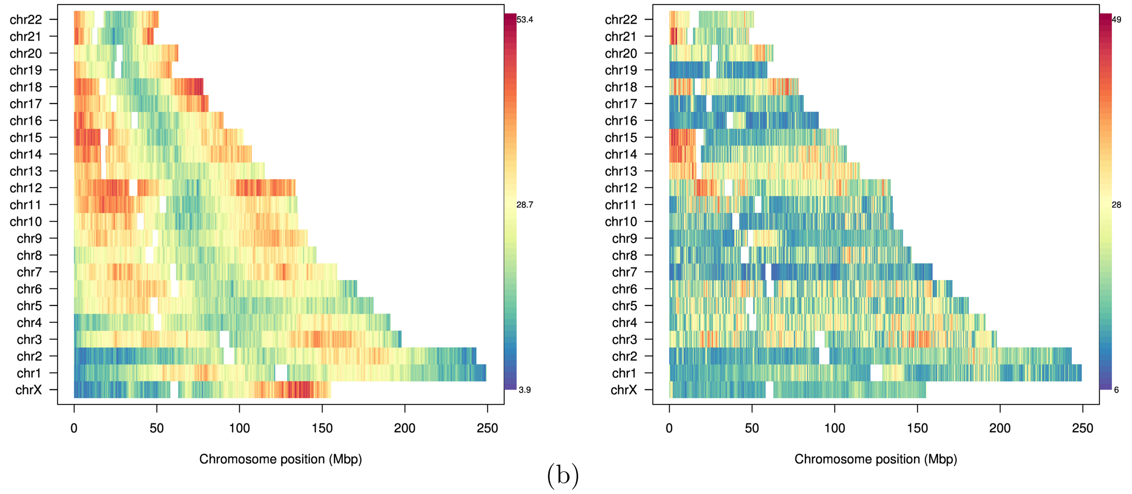


(a) (b)

**Supplementary Figure S7**: **Genome-wide variability of radial bead position.** The panels are based on configurations obtained starting from the phenomenological prepositioning of the chromosomes in Bolzer *et al.* (2005), immediately prior to (a) and after (b) applying the steering protocol appropriate for hESC cell line based on the analysis of the data in Dixon *et al* (2012). Numbers indicate the standard deviation of the radial position across the 10 replicate simulations.

**Embryonic stem cells (hESC) nuclei with random prepositioning of the chromosomes**

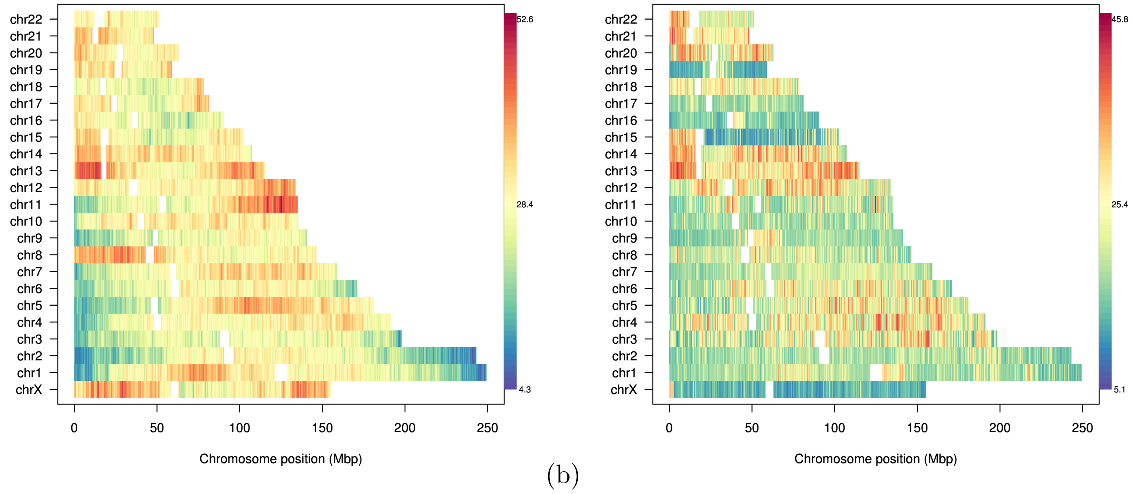


(a) (b)

**Supplementary Figure S8**: **Genome-wide variability of radial bead position.** The panels are based on configurations obtained starting from a random prepositioning of the chromosomes, immediately prior to (a) and after (b) applying the steering protocol appropriate for hESC cell line based on the analysis of the data in Dixon *et al* (2012). Numbers indicate the standard deviation of the radial position across the 10 replicate simulations.

## Lung fibroblasts (IMR90) nuclei with phenomenological prepositioning of the chromosomes

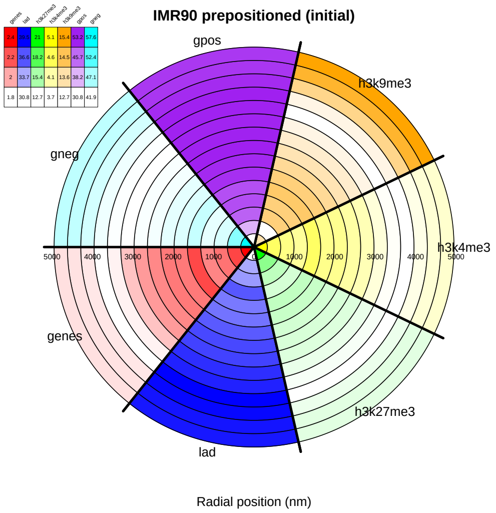


(a)

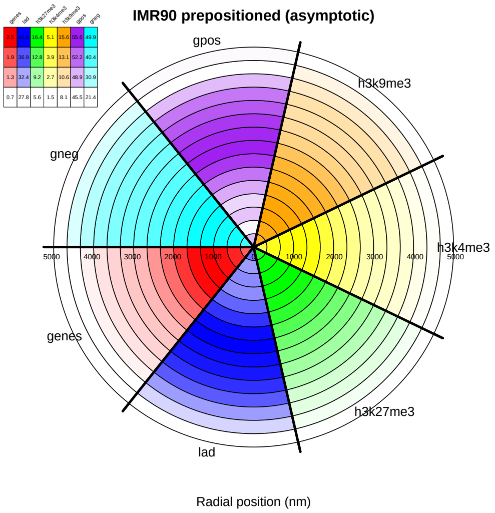


(b)

**Supplementary Figure S9**: **Nuclear positioning of functionally-oriented genomic regions.** Circular histograms giving the relative density (in percent) based on H3K9me3 (orange), H3K4me3 (yellow), H3K27me3 (green), LADs (blue) and genes (red), and negative (cyan) and positive (purple) Giemsa staining bands. Circle slices (thickness $\sim$ 320nm) indicate radial position (in nm) within the nucleus aggregated across all 10 replicate simulations, and the numbers indicate the percentage of beads associated with the given feature relative to the total number of beads in the given circle slice. The figure is based on configurations obtained starting from the phenomenological prepositioning of the chromosomes in Bolzer *et al.* (2005), immediately prior to (a) and after (b) applying the steering protocol appropriate for IMR90 cell line based on the analysis of the data in Dixon *et al* (2012).

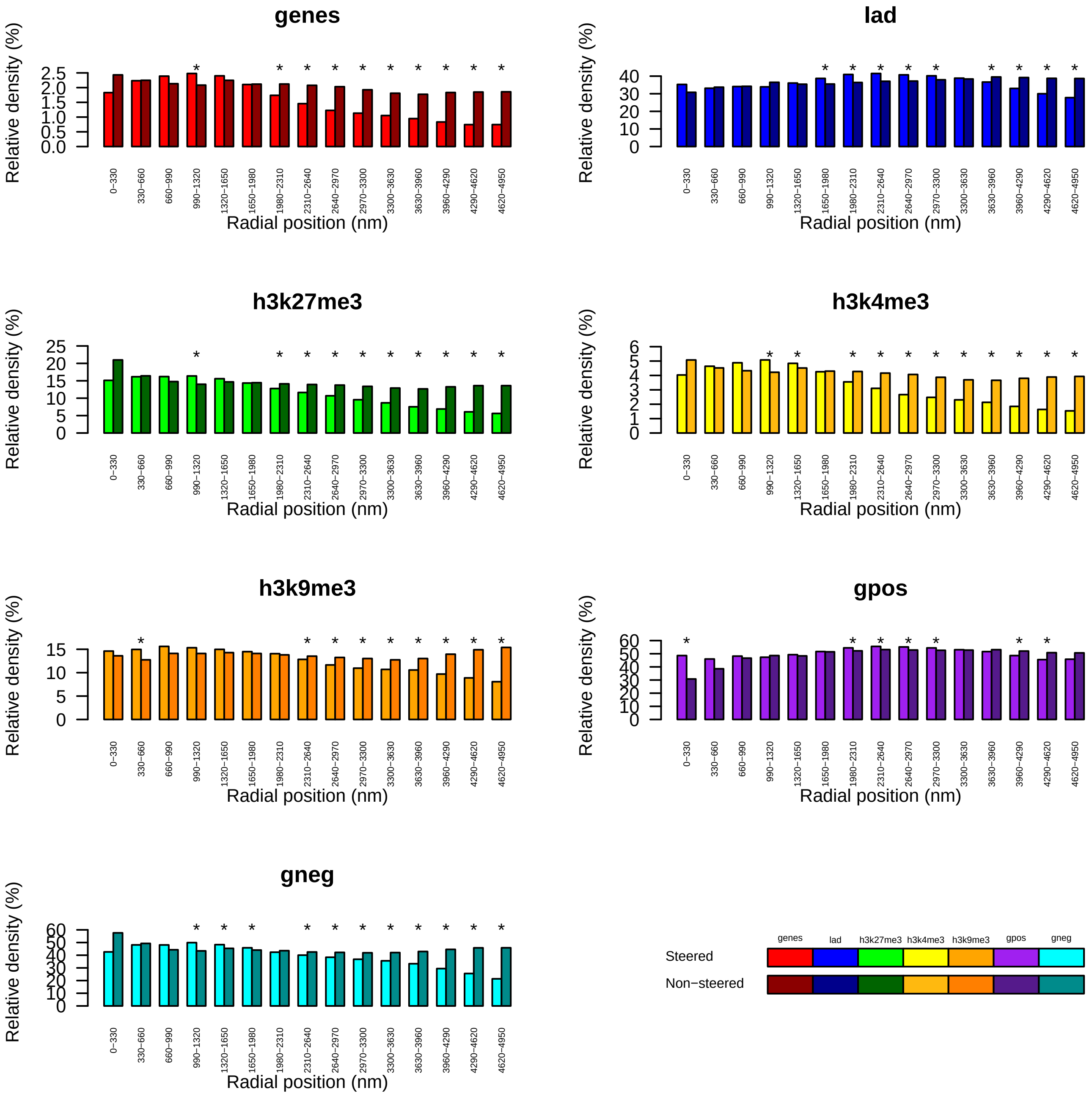


**Supplementary Figure S10**: **Histograms of the relative density of genes, LADs, H3K27me3, H3K4me3, H3K9me3, and positive (gpos) and negative (gneg) Giemsa staining bands in concentric equally thick radial shells of the nucleus.** The figure is based on configurations obtained starting from the phenomenological prepositioning of the chromosomes in Bolzer *et al.* (2005), immediately prior to (Non-steered) and after (Steered) applying the steering protocol appropriate for IMR90 cell line based on the analysis of the data in Dixon *et al* (2012). The asterisks indicate statistically significant differences between the two cases, using the Wilcoxon test with a p-value cutoff of 0.05. The plots complement the information provided in Fig. 4 of the main text.

## Lung fibroblasts (IMR90) nuclei with random prepositioning of the chromosomes

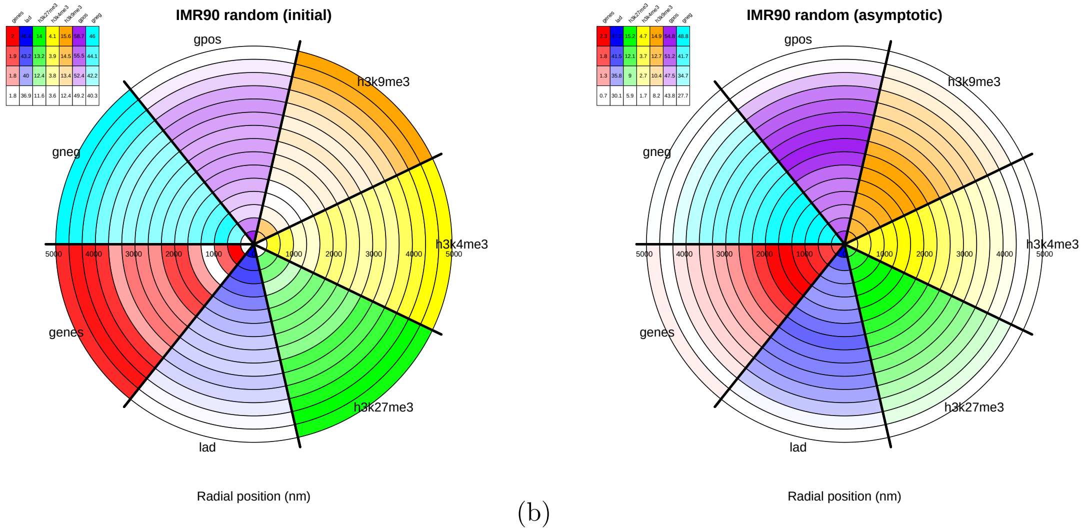


(a) (b)

**Supplementary Figure S11**: **Nuclear positioning of functionally-oriented genomic regions.** Circular histograms giving the relative density (in percent) based on H3K9me3 (orange), H3K4me3 (yellow), H3K27me3 (green), LADs (blue) and genes (red), and negative (cyan) and positive (purple) Giemsa staining bands. Circle slices (thickness $\sim$ 320nm) indicate radial position (in nm) within the nucleus aggregated across all 10 replicate simulations, and the numbers indicate the percentage of beads associated with the given feature relative to the total number of beads in the given circle slice. The figure is based on configurations obtained starting from a random prepositioning of the chromosomes, immediately prior to (a) and after (b) applying the steering protocol appropriate for IMR90 cell line based on the analysis of the data in Dixon *et al* (2012).

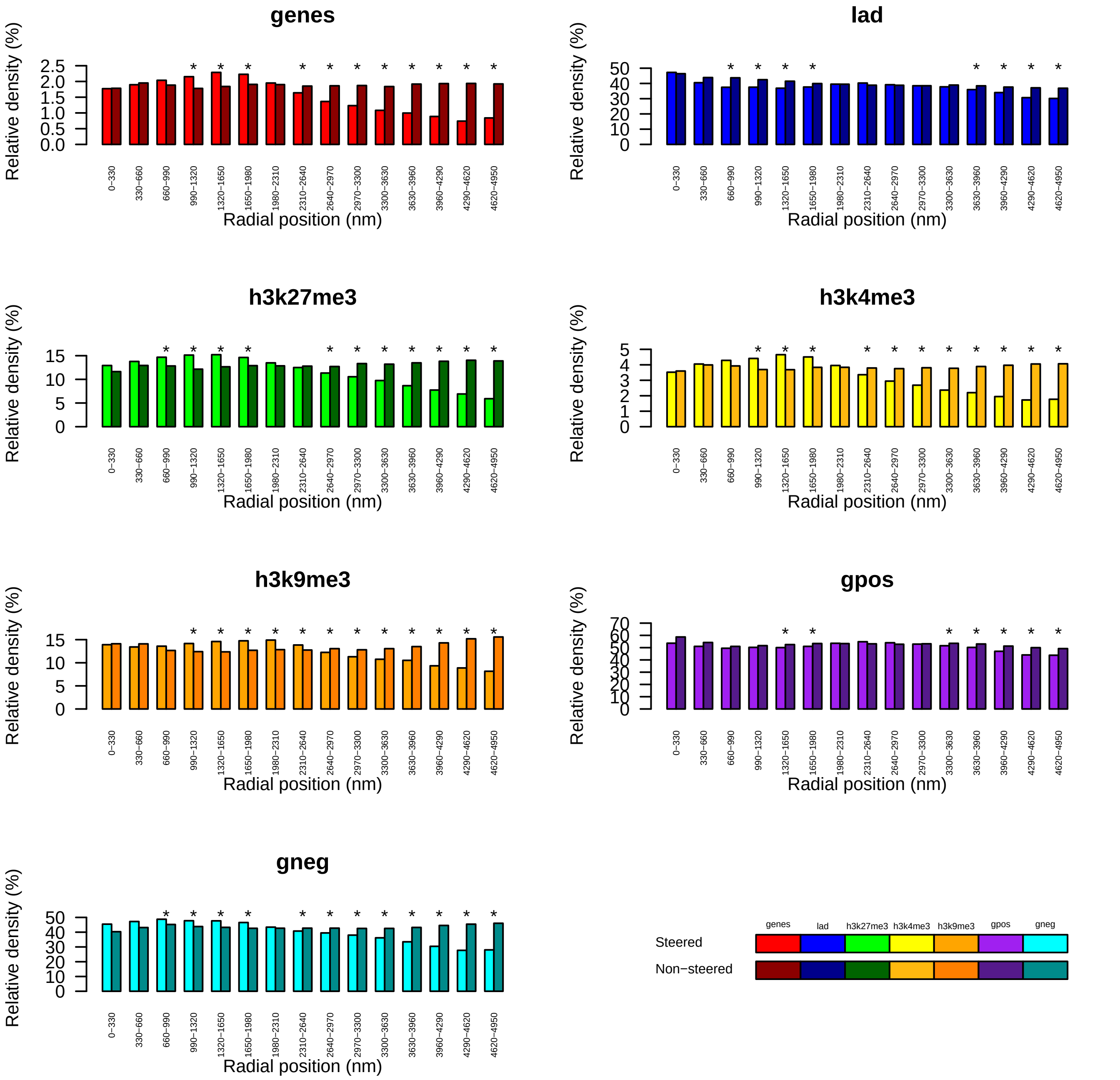


**Supplementary Figure S12**: **Histograms of the relative density of genes, LADs, H3K27me3, H3K4me3, H3K9me3, and positive (gpos) and negative (gneg) Giemsa staining bands in concentric equally thick radial shells of the nucleus.** The figure is based on configurations obtained starting from a random prepositioning of the chromosomes, immediately prior to (Non-steered) and after (Steered) applying the steering protocol appropriate for IMR90 cell line based on the analysis of the data in Dixon *et al* (2012).

**Embryonic stem cells (hESC) nuclei with phenomenological prepositioning of the chromosomes**

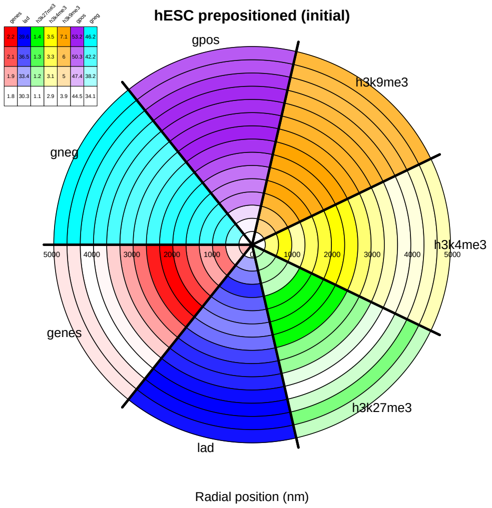


(a)

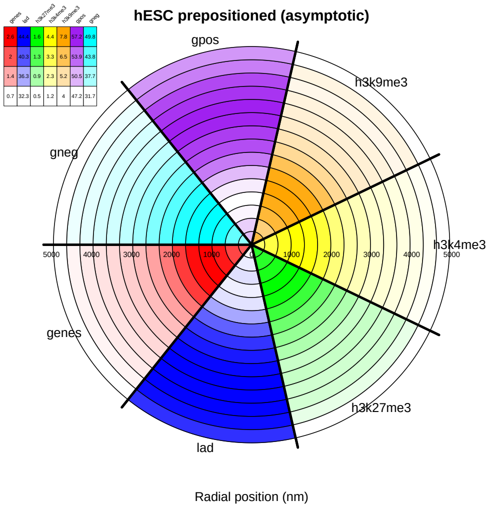


(b)

**Supplementary Figure S13**: **Nuclear positioning of functionally-oriented genomic regions.** Circular histogram giving the relative density (in percent) based on H3K9me3 (orange), H3K4me3 (yellow), H3K27me3 (green), LADs (blue) and genes (red), and negative (cyan) and positive (purple) Giemsa staining bands. Circle slices (thickness $\sim$ 320nm) indicate radial position (in nm) within the nucleus aggregated across all 10 replicate simulations, and the numbers indicate the percentage of beads associated with the given feature relative to the total number of beads in the given circle slice. The figure is based on configurations obtained starting from the phenomenological prepositioning of the chromosomes in Bolzer *et al.* (2005), immediately prior to (a) and after (b) applying the steering protocol appropriate for hESC cell line based on the analysis of the data in Dixon *et al* (2012).

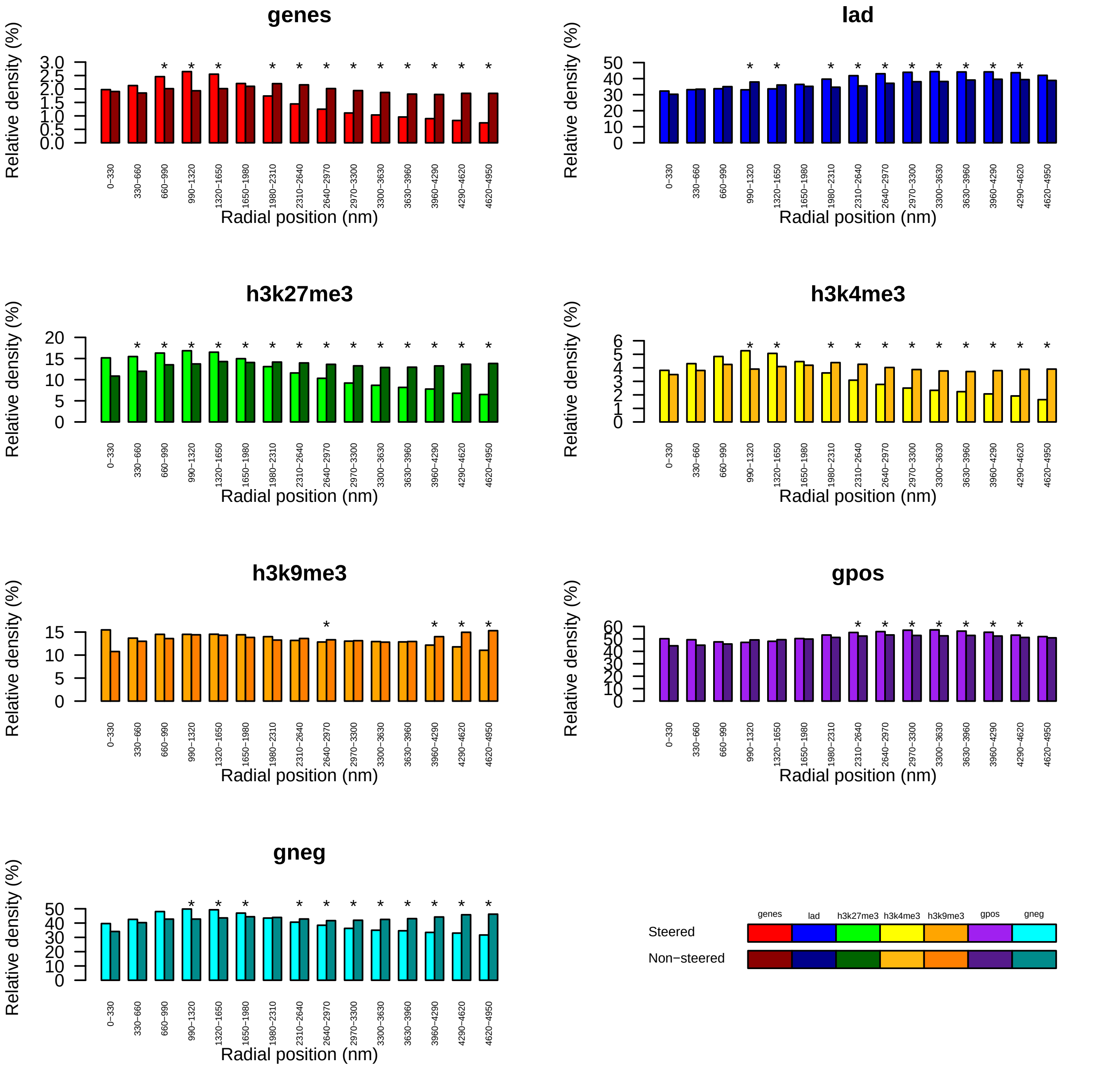


**Supplementary Figure S14**: **Histograms of the relative density of genes, LADs, H3K27me3, H3K4me3, H3K9me3, and positive (gpos) and negative (gneg) Giemsa staining bands in concentric equally thick radial shells of the nucleus.** The figure is based on configurations obtained starting from the phenomenological prepositioning of the chromosomes in Bolzer *et al.* (2005), immediately prior to (Non-steered) and after (Steered) applying the steering protocol appropriate for hESC cell line based on the analysis of the data in Dixon *et al* (2012).

**Embryonic stem cells (hESC) nuclei with random prepositioning of the chromosomes**

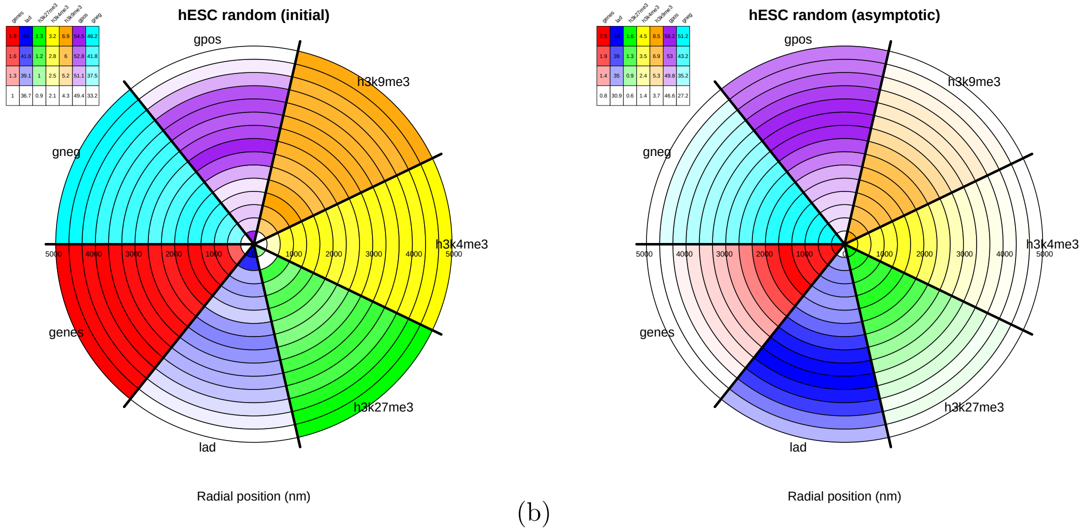


(a) (b)

**Supplementary Figure S15**: **Nuclear positioning of functionally-oriented genomic regions.** Circular histogram giving the relative density (in percent) based on H3K9me3 (orange), H3K4me3 (yellow), H3K27me3 (green), LADs (blue) and genes (red), and negative (cyan) and positive (purple) Giemsa staining bands. Circle slices (thickness $\sim$ 320nm) indicate radial position (in nm) within the bounding nucleus aggregated across all 10 replicate simulations, and the numbers indicate the percentage of beads associated with the given feature relative to the total number of beads in the given circle slice. The figure is based on configurations obtained starting from a random prepositioning of the chromosomes, immediately prior to and after applying the steering protocol appropriate for hESC cell line based on the analysis of the data in Dixon *et al* (2012).

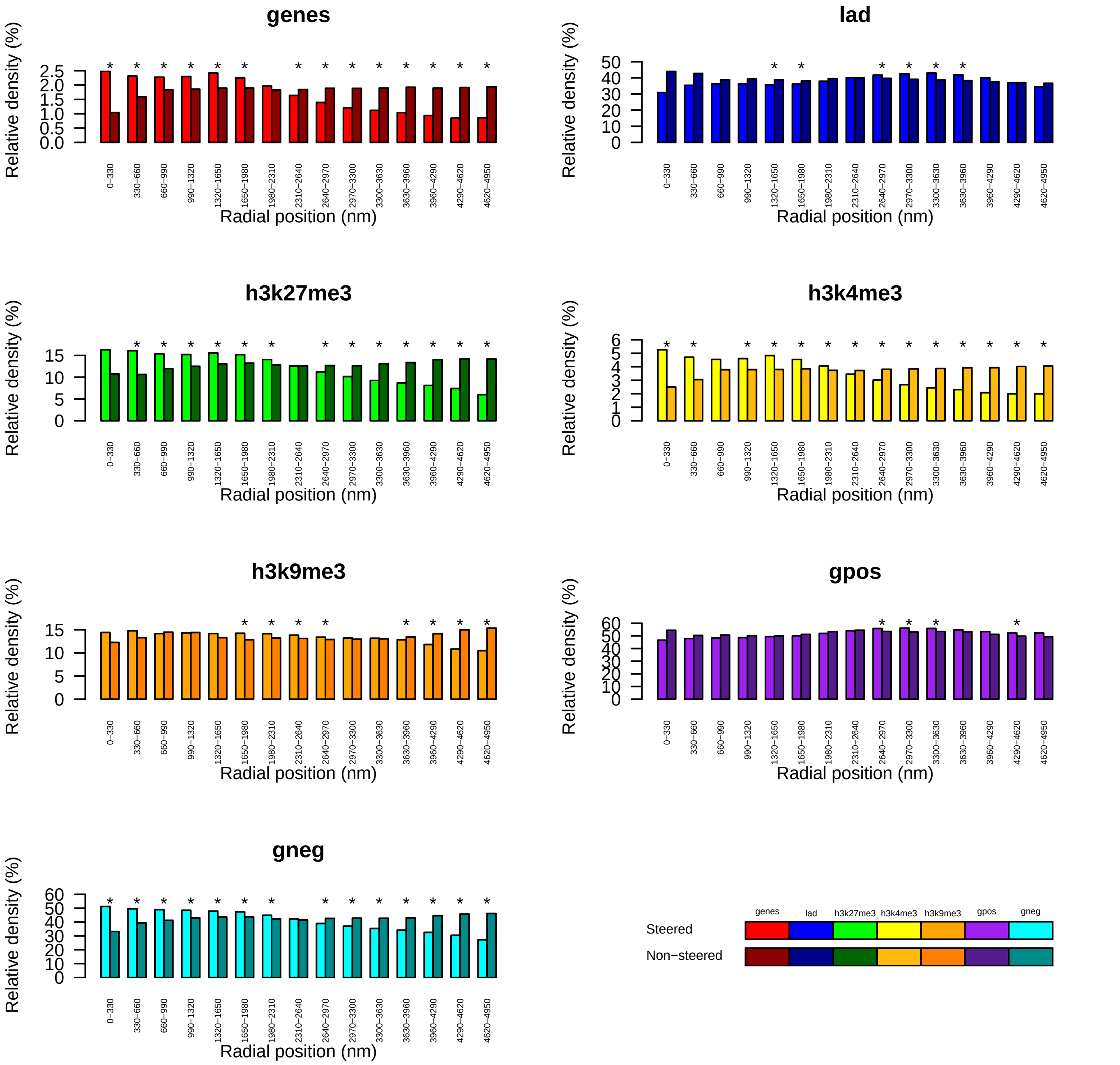


**Supplementary Figure S16**: **Histograms of the relative density of genes, LADs, H3K27me3, H3K4me3, H3K9me3, and positive (gpos) and negative (gneg) Giemsa staining bands in concentric equally thick radial shells of the nucleus.** The figure is based on configurations obtained starting from a random prepositioning of the chromosomes, immediately prior to (Non-steered) and after (Steered) applying the steering protocol appropriate for hESC cell line based on the analysis of the data in Dixon *et al* (2012).

**Lung fibroblasts (IMR90) nuclei with phenomenological prepositioning of the chromosomes**

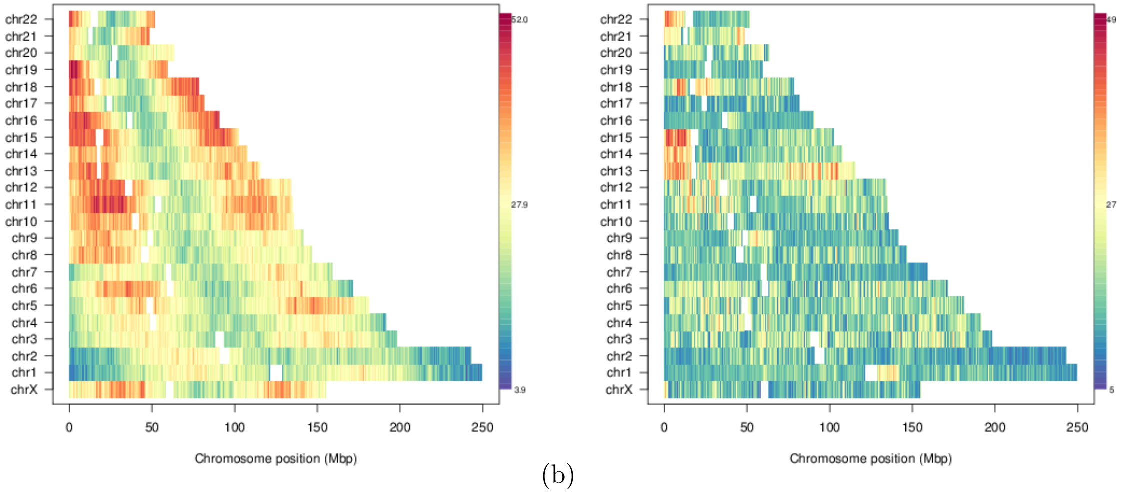


(a) (b)

**Supplementary Figure S17**: **Genome-wide variability of radial bead position.** The panels are based on configurations obtained starting from the phenomenological prepositioning of the chromosomes in Bolzer *et al.* (2005), immediately prior to any steering (a) and after (b) applying the steering additional steering of the target pairs in Rao *et al* (2014). Numbers indicate the standard deviation of the radial position across the 10 replicate simulations.

**Lung fibroblasts (IMR90) nuclei with phenomenological prepositioning of the chromosomes**

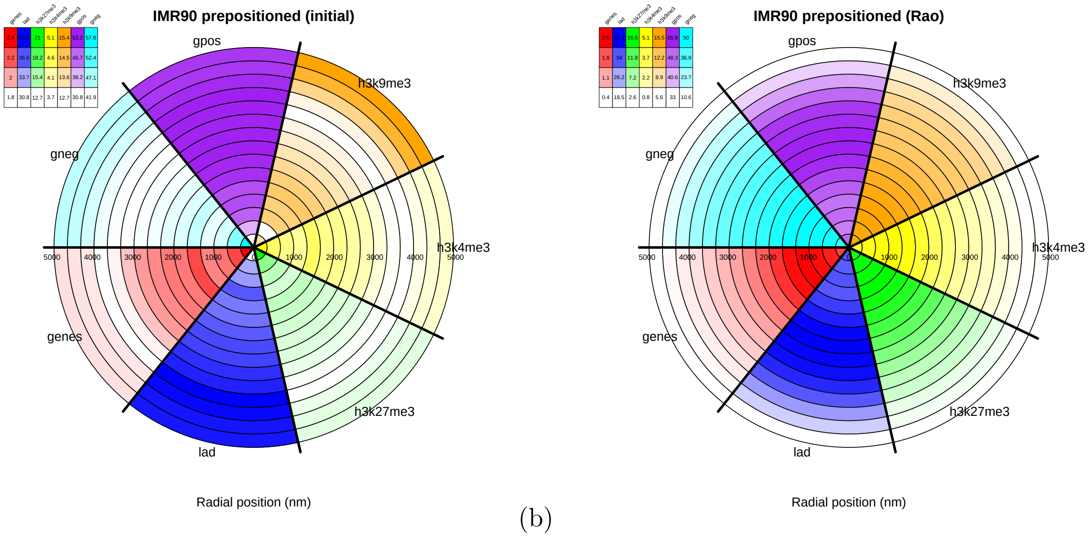


(a) (b)

**Supplementary Figure S18**: **Nuclear positioning of functionally-oriented genomic regions.** Circular histograms giving the relative density (in percent) based on H3K9me3 (orange), H3K4me3 (yellow), H3K27me3 (green), LADs (blue) and genes (red), and negative (cyan) and positive (purple) Giemsa staining bands. Circle slices (thickness $\sim$ 320nm) indicate radial position (in nm) within the nucleus aggregated across all 10 replicate simulations, and the numbers indicate the percentage of beads associated with the given feature relative to the total number of beads in the given circle slice. The figure is based on configurations obtained starting from the phenomenological prepositioning of the chromosomes in Bolzer *et al.* (2005), immediately prior to any steering (a) and after (b) applying the additional steering of the target pairs in Rao *et al* (2014).

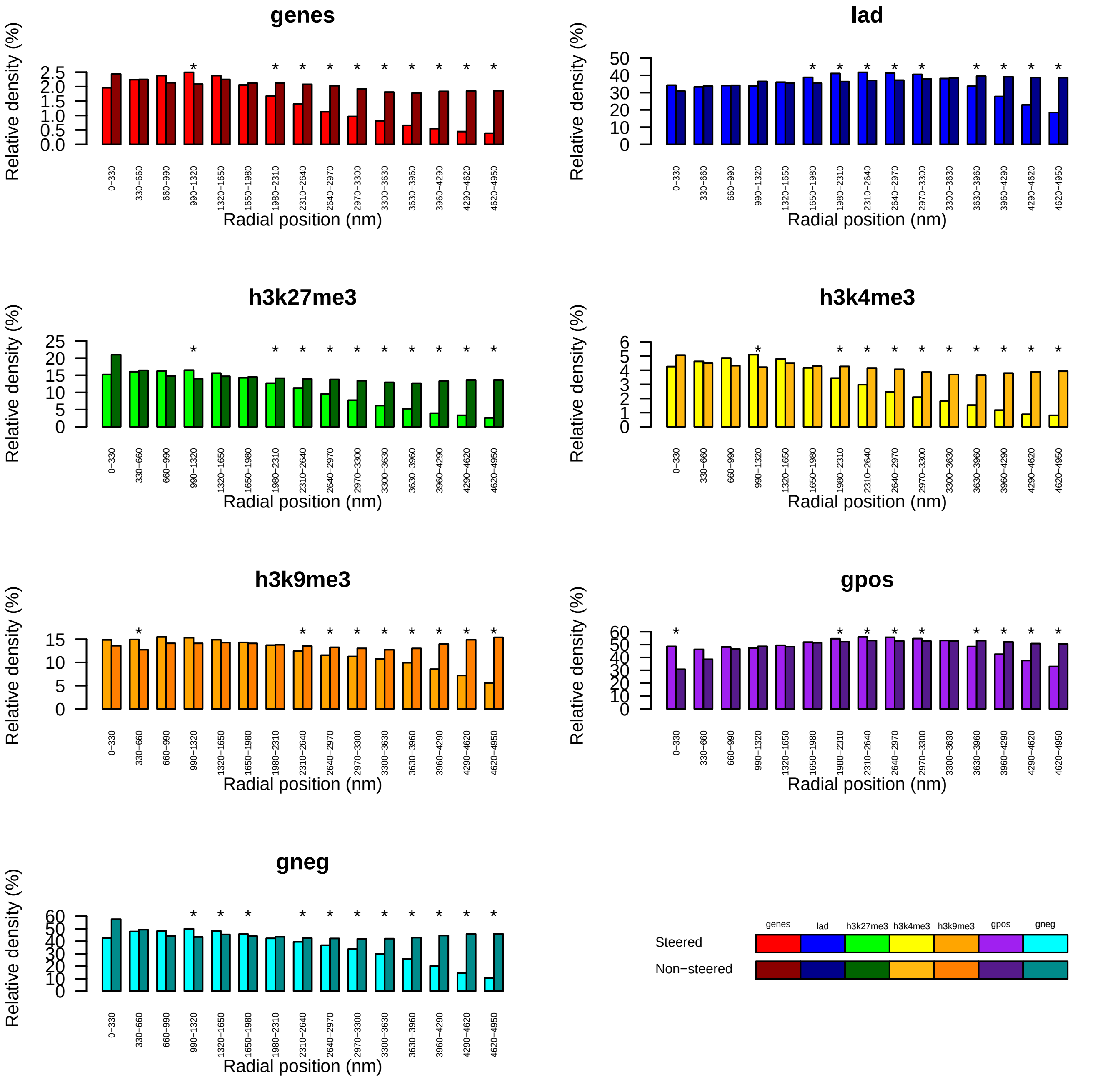


**Supplementary Figure S19**: **Histograms of the relative density of genes, LADs, H3K27me3, H3K4me3, H3K9me3, and positive (gpos) and negative (gneg) Giemsa staining bands in concentric equally thick radial shells of the nucleus.** The figure is based on configurations obtained starting from the phenomenological prepositioning of the chromosomes in Bolzer *et al.* (2005), immediately prior to any steering (Non-steered) and after applying the additional steering of the target pairs in Rao *et al* (Steered). The asterisks indicate statistically significant differences between the two cases, using the Wilcoxon test with a p-value cutoff of 0.05.

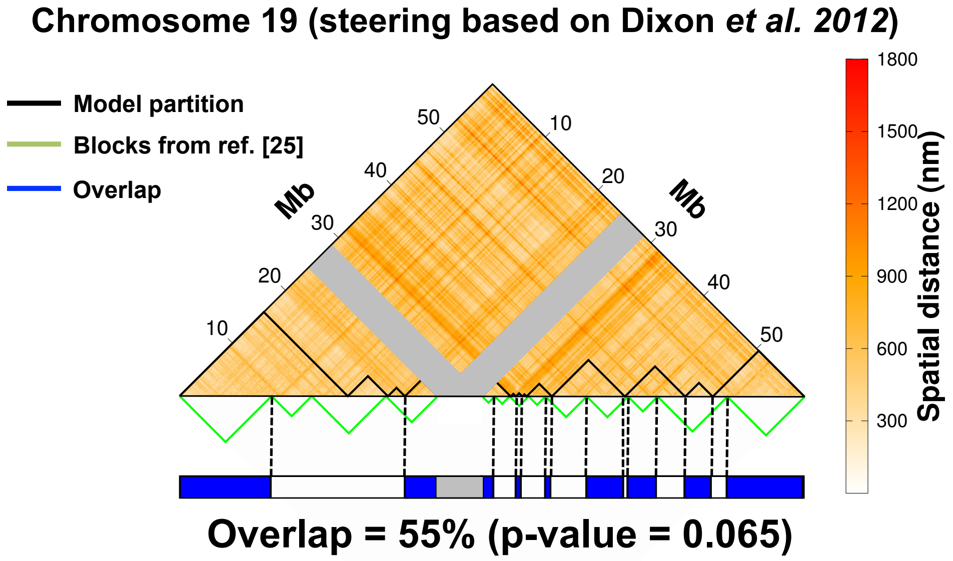


**Supplementary Figure S20**: **Structural macrodomain organization.** The upper triangle of the map of average spatial distance (100kb resolution) of chromosome 19 at the end of the steering dynamics based on the significant target contacts obtained from Dixon *et al* (2012) is shown. The gray bands mark the centromeric region. The boundaries of the 13 spatial macrodomains, identified with a clustering analysis of the distance matrix (see Methods), are overlaid on the map and the boundaries of the spatial *blocks* in Kalhor *et al.* (2012) are shown below. The consistency of the two partitions is visually conveyed in the chromosome cartoon at the bottom. Overlapping regions, shown in blue, account for 55% of the chromosome (centromere excluded).
