# Supplementary material for: Hi-C-constrained physical models of human chromosomes recover functionally-related properties of genome organization
Source: Sci Rep. 2016 Oct 27;6:35985. doi: 10.1038/srep35985 (PMC5081523; doi:10.1038/srep35985)
Supplement: Supplementary Methods [file srep35985-s2.pdf]

Supplementary Methods

"Hi-C-constrained physical models of human chromosomes recover functionally-related properties of genome organization"

by Marco Di Stefano, Jonas Paulsen, Tonje G. Lien, Eivind Hovig, and Cristian Micheletti

MODELING CHROMOSOME STRUCTURE AND DYNAMICS

The feasibility to establish simultaneously the significant Hi-C contacts was explored by using model chromosomes and steered molecular dynamics simulations analogous to those used in ref. [1] and which are further detailed below.

The chromosome polymer model

Each chromosome was modelled using a general bead-spring model [2]:

$$\mathcal{H} = U_{\mathrm{LJ}} + U_{\mathrm{FENE}} + U_{\mathrm{KP}}. \tag{1}$$

The first term is a truncated and shifted, purely repulsive Lennard-Jones potential:

$$U_{LJ}(i,j) = \begin{cases} 4k_BT\epsilon_{ij}\left[\left(\frac{\sigma}{d_{i,j}}\right)^{12} - \left(\frac{\sigma}{d_{i,j}}\right)^{6} + 1/4\right] & \text{if } d_{i,j} \leq 2^{1/6}\sigma, \\ 0 & \text{if } d_{i,j} > 2^{1/6}\sigma. \end{cases} \tag{2}$$

where k_B is the Boltzmann constant, T the temperature, ϵ_{ij} is equal to 10 if $|i-j|=1$, and 1 otherwise, $\sigma = 30$nm is the thickness of the chain and $d_{i,j}$ is the modulus of $\vec{d}_{i,j} = \vec{r}_i - \vec{r}_j$ which is the distance vector between monomers i and j at positions $\vec{r}_i$ and $\vec{r}_j$, respectively. This term controls the *cis*-chain excluded volume interaction.
The second term is a FENE potential:

$$U_{FENE}(i,i+1) = -150k_BT\left(\frac{R_0}{\sigma}\right)^2\left[1 - \left(\frac{d_{i,i+1}}{R_0}\right)^2\right] \tag{3}$$

where $R_0 = 1.5\sigma$ is the maximum bond length. This term ensures the connectivity between consecutive beads of the same polymer chain. The combined action of the FENE and LJ potential between consecutive beads is such that, during the free and steered simulations, the average bonds length is close to σ and never exceeds 1.3σ.
The third term is a Kratky-Porod, or bending potential:

$$U_{br}(i,i+1,i+2) = \frac{k_BTl_p}{\sigma}\left(1 - \frac{\vec{d}_{i,i+1}\cdot\vec{d}_{i+1,i+2}}{d_{i,i+1}d_{i+1,i+2}}\right), \tag{4}$$

where the chain persistence length, l_p, has been set equal to $5\sigma = 150$nm to reproduce the experimental rigidity of the chromatin fiber [3].

Different chromosomes interact only via excluded volume interactions, through the LJ repulsion of their constitutive beads.

Description of the free chain dynamics

The free dynamics of the chains was described with an underdamped Langevin equation, while the steering process was guided by using pairwise harmonic constraints. In both cases the dynamics was integrated with the LAMMPS simulation package [4].

Specifically, the underdamped Langevin equation is:

$$m\ddot{r}_{i\alpha} = -\partial_{i\alpha}\mathcal{H} - \gamma\dot{r}_{i\alpha} + \eta_{i\alpha}(t) \tag{5}$$

where is the m is the bead mass which was set equal to the LAMMPS default value, $\mathcal{H}$ is the system energy in Eq. 1, the index i runs over all the particles in the system, and $\alpha = (x, y, z)$ indicates the Cartesian components. The stochastic noise term $\eta_{i\alpha}(t)$ satisfies the standard fluctuation dissipation conditions: $\langle\eta_{i\alpha}\rangle = 0$ and $\langle\eta_{i\alpha}(t)\,\eta_{j\beta}(t')\rangle = 2\kappa_B T\gamma\,\delta_{ij}\delta_{\alpha\beta}\delta(t-t')$, where $\gamma = 0.5\tau_{LJ}^{-1}$ is the friction coefficient, $\tau_{LJ} = \sigma(m/\epsilon)^{1/2}$ is the Lennard-Jones time, δ_{ij} is the Kronecher delta, and $\delta(t-t')$ is the Dirac delta.

The integration time step used in the LAMMPS numerical integration of the Langevin was equal to $\Delta t = 0.006\tau_{LJ}$.

Steered molecular dynamics protocol

The colocalization of the target pairs of 100 *kbp*-long chromosome stretches was promoted by using a steered molecular dynamics protocol that progressively favoured the spatial proximity of the target pairs in each model chromosome.

Specifically, we mapped each pair of selected regions, A and B, onto the corresponding 33beads-long stretches of the chromosome chain and added to the system energy an harmonic constrain:

$$U_{\mathrm{H}} = \frac{1}{2}k\,(L,t)\,(d_{A,B} - d_0/2.0)^2 \tag{6}$$

where $d_{A,B}$ is the distance of the centers of mass of the chromosome stretches and the equilibrium distance is set to half of the contact distance $d_0 = 120$nm. The stiffness of the harmonic constraint was controlled by a spring constant $k\,(L,t)$ depending on the sequence separation between the two regions, L, and the simulation time, t.

The sequence-separation dependence of k was introduced to ensure that the steering process is not dominated by the target pairs at the largest sequence separation. To this purpose, we made the spring constant dependent on the sequence separation L of the target pairs so that, in the decondensed state (which is the state of the system just before the steering process takes place) all pairs are pulled together with the same average force, irrespective of their sequence separation.

To accomplish this balancing of the spring constant, we used a statistical reweighting approach. Specifically, we computed the distribution of the square spatial distances between all pairs of 100kbp-long chromosome stretches. We subdivided them in 25 groups with the first, second, etc. group gathering pairs at genomic distances, L, in the $0-10$Mbp, $10-20$Mbp ranges, etc. Within each group, we computed the normalized distribution of the spatial distances, d, between al the pairs of 100kbp chromosome strands in the conformation of the decondensed chromosome system. Each of the 6 obtained distributions was fitted with the function:

$$y = A \cdot x^2 \cdot \exp\left(-\frac{x^2}{2\,\sigma^2}\right) \tag{7}$$

which takes into account the radial weight. The distance distribution and the Gaussian fit for bin 1 is shown in Supplementary Figure 1A.

The gathered distance statistics was next reweighted with a Gaussian function:

$$w\,(k) = e^{-k/2(d-d_0/2)^2}$$

where $d_0 = 120$nm is the cutoff distance for defining a contact between target pairs. By doing so, we found the value of the spring constant k to yield at least 90.0% of the pairwise distances below the contact radius. The re-weighted probability distribution for bin 1 is shown in Supplementary Figure 1B.

For robustness, the spring constant of pairs at separations larger than 60Mb (i.e. appreciably beyond the average chromosome length) was set equal to the same one of the pairs at distances in the 50-60Mb range The obtained values of the reference spring constants are shown in Supplementary Table I for each bin.

At the beginning of the simulation, the spring constants are set to 10% of these reference values and then are progressively increased.

The simultaneous application of the N_{tp} constraints to each chromosome was implemented by using the PLUMED plugin for LAMMPS [5]. The spring constants were gradually ramped linearly every $6.0\tau_{LJ}$ of steered simulation, so

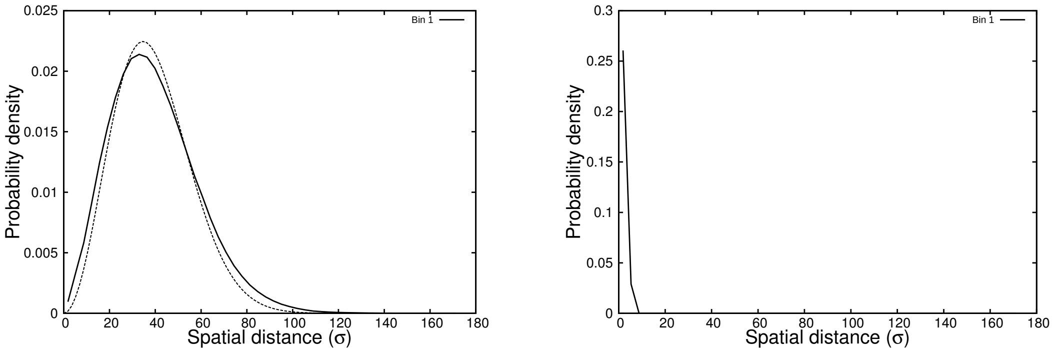

A B

Figure M1: Spatial distances probability density.

Bin	Genomic separation (Mb)	k
1	0-10	0.67714
2	10-20	0.49177
3	20-30	0.27520
4	30-40	0.24356
5	40-50	0.24399
6	50-60	0.22315
7-25	60-250	0.22315

Table SI: Spring constant.

to avoid driving the system significantly out of equilibrium: $k\,(L,t) = k(L,0)t/(6.0\tau_{LJ})$ for each value of L. Moreover, the resulting pulling force between each constrained pair was controlled every $6.0\tau_{LJ}$ and, if it exceeded a maximum pulling force of $300\,\epsilon/\sigma$, we set it to this maximum value. This maximum corresponds to the nominal magnitude of the bonding force (LJ+FENE) of the pairs of nearest neighbor beads at a distance of 1σ.

This maximum force was low enough and the simulation time-step Δt short enough to avoid appreciable over-stretching of the bond connecting the beads, as this may result in unphysical passages of the strands through each other during a numerical integration time step.

The chromosome pre-mitotic re-condensation

After steering, the model chromosomes were reconfigured to a linear, pre-mitotic state. This was done by switching off the harmonic restraints of the target contacts and by replacing them with restraints between pairs of *loci* at the regular sequence separation of 200 kilo-bases. The new target constraints are applied using harmonic springs having the same coupling constant equal to $2\epsilon/\sigma^2$ during the entire simulation span. The equlibrium distance of the springs is, instead, decreased in steps of 30 nm every 0.6 τ_{LJ} from 200 nm (the maximum extension of a 200 kb chromatin strand) to 30 nm (the size of a bead). At the last equilibrium distance, the simulation is prolungated up to 300 τ_{LJ}. This procedure results in the reconfiguration of the chromosomes into a succession of 200 kb loops arranged in a string-like fashion. This target arrangement is analogous to the mitotic or linear chromosome models of refs. [6, 7]. This re-condesation procedure is applied to the 10 replicates of the human embryonic stem cells (hESC) system before and after steering.

Calculation of the contact distance for target pairs.

The bending properties of a polymer chain has a large impact on the probability of looping, and hence, on bringing to regions of the chain in spatial contact. The parameter that tunes this property in the model used in this study is the persistence length (see Eq.4), which for a worm-like chain is half the Kuhn-length, l_K, of the polymer chain.
To determine the contact radius for two constrained chromosome stretches in a more general case (non-Gaussian chains), we considered the expression of the mean square gyration radius R_g^2, which has been established by Benoit and Doty in ref. [8] for a worm-like chain of contour length L_c, which spans M Kuhn-lengths ($M = L_c/l_K$):

$$\left\langle R_g^2\left(M\right)\right\rangle = \frac{M\,l_k^2}{6} - \frac{l_k^2}{4} + \frac{l_k^2}{4\,M} - \frac{l_k^2}{8\,M^2}\left(1 - e^{-2M}\right) \tag{8}$$

A heuristic use of expression (8) is to estimate the effective size of the region occupied in equilibrium by portions of contour length L_c from a long polymer chain. We therefore consider the occupied region to be spherical, centred on the centre of mass of the segment, and with a radius equal to $\sqrt{\left\langle R_g^2\left(M\right)\right\rangle}$. The criterion to define an established spatial contact between a pair of segments of contour length L_c should be based on the overlap volume of the two spheres spanned separately by the two stretches.
The volume of the intersection between two spheres of identical radius R as a function of the the distance d between the centres of the sphere is (see `http://mathworld.wolfram.com/Sphere-SphereIntersection.html`):

$$V = \frac{1}{12}\pi\left(4R + d\right)\left(2R - d\right)^2$$

As a criterion for a significant overlap, we consider the threshold of 50% of the volume of each individual sphere. The corresponding sphere distance (contact radius) must be equal to:

$$d \;=\; 0.694592710\,R$$

For the chromosome chains studied here, the contour length L_c of the stretches to co-localize accounts for 100kbp which map onto $\sim 1\mu m$ [9, 10]. Given the Kuhn-length of the chromosome fiber $l_K = 300\,nm$ [9, 10], M results to be equal to ~ 3.3. This corresponds to a contact distance of about:

$$d = 0.694592710\sqrt{\left\langle R_g^2\left(3.3\right)\right\rangle} \sim 120nm$$

[1] M. Di Stefano, A. Rosa, V. Belcastro, D. di Bernardo, and C. Micheletti, PLoS Comput. Biol. **9**, e1003019 (2013).
[2] K. Kremer and G. S. Grest, J. Chem. Phys. **92**, 5057 (1990).
[3] K. Bystricky, P. Heun, L. Gehlen, J. Langowski, and S. M. Gasser, Proc. Natl. Acad. Sci. USA **101**, 16495 (2004).
[4] S. Plimpton, J. Comp. Phys. **117**, 1 (1995).
[5] M. Bonomi, D. Branduardi, G. Bussi, C. Camilloni, D. Provasi, P. Raiteri, D. Donadio, F. Marinelli, F. Pietrucci, R. A. Broglia, et al., Comp. Phys. Comm. **180**, 1961 (2009).
[6] J. Sikorav and G. Jannink, Biophys. J. **66**, 827 (1994).
[7] N. Naumova, M. Imakaev, G. Fudenberg, Y. Zhan, B. R. Lajoie, L. A. Mirny, and J. Dekker, Science **342**, 948 (2013).
[8] H. Benoit and P. Doty, The Journal of Physical Chemistry **57**, 958 (1953).
[9] A. Rosa and R. Everaers, PLoS Comput. Biol. **4**, e1000153 (2008).
[10] A. Rosa, N. B. Becker, and R. Everaers, Biophys. J. **98**, 2410 (2010).
